# Monitoring Web Site Usage of e-Bug: A Hygiene and Antibiotic Awareness Resource for Children

**DOI:** 10.2196/resprot.4049

**Published:** 2015-11-13

**Authors:** Vicki L Young, Vijayamaharaj Rajapandian, Charlotte V Eley, Beverley A Hoekstra, Donna M Lecky, Cliodna AM McNulty

**Affiliations:** ^1^ Public Health England Primary Care Unit Gloucester United Kingdom

**Keywords:** e-Bug, educational resources, public health, antibiotics

## Abstract

**Background:**

e-Bug is an educational resource which teaches children and young people about microbes, hygiene, infection, and prudent antibiotic use. The e-Bug resources are available in over 22 different languages and they are used widely across the globe. The resources can be accessed from the e-Bug website.

**Objective:**

The objective of this study was to analyze the usage of the e-Bug website in order to understand how users access the website, where and when they access the site, and to review variation in use across the different areas of the site.

**Methods:**

The usage statistics for the e-Bug website were monitored by Google Analytics between September 2010 and August 2013.

**Results:**

The statistics show the website had over 324,000 visits during the three years, from just under 250,000 visitors, with the number of visitors increasing year after year. Visitors accessed the website from 211 different countries, with more than 267,000 documents downloaded. The majority of visitors were from the United Kingdom and visited the English website, although countries such as France and Portugal were also frequent visitors.

**Conclusions:**

These website statistics confirm that e-Bug is frequently used across Europe and highlight that e-Bug use has expanded across the world. The findings from this report will be used to inform future modifications or updates to the materials, as well as the development of new educational resources.

## Introduction

### Controlling Antimicrobial Resistance

Antimicrobial resistance is becoming increasingly recognized worldwide as a threat to public health. A recent World Health Organization (WHO) report on antimicrobial resistance highlights the “alarming levels” of bacterial resistance seen in many parts of the world, with situations where many of the available treatment options for common infections are becoming ineffective [1]. Education of the public and professionals on the importance of controlling antimicrobial resistance through reducing antibiotic use remains a key focus of the European Centre for Disease Prevention and Control, through their annual European Antibiotic Awareness Day (EAAD) campaign, and the UK’s Department of Health, through their Five Year Antimicrobial Resistance Strategy [2]. The WHO is also urging member states to strengthen drug management systems and to support research into new treatment options [3].

The importance and effectiveness of health education websites have been demonstrated previously. A survey by the Pew Internet & American Life Project in 2009 [4] revealed that 8 out of 10 Internet users search on the Internet for health information. In addition, an analysis of the Children First for Health website revealed that 30% of inquiries regarding health topics were received directly from children and young people less than 19 years of age [5].

The Internet is a suitable tool for health promotion [6] and Internet health interventions have been shown to change behavior [7,8]. A study by Yardley et al [9] demonstrated that a Web based health intervention can lead to an increase in hand washing; suggesting hygienic behavior can be influenced through Internet tools. Evidence from the National Health Service (NHS) England annual review (2013-2014) supports that the Internet is an appropriate method for health promotion and that the Internet can be used to reach a wide target audience. The website NHS Choices has become the largest European health and care website aimed at the public, with around 27 million visitors per month [10].

### The e-Bug Educational Resource

e-Bug is an educational resource for young people in the school and home setting, which aims to help reduce antimicrobial use across Europe. Children are our future generation of antibiotic users, gatekeepers, and prescribers, and education on different types of microbes and prudent antibiotic use instills these important messages at a young age. In addition, by education on key hygiene messages, such as how bacteria are spread from person to person, e-Bug aims to reduce the spread of infection through the community and hence reduce antibiotic use through this mechanism.

The e-Bug resource is led by Public Health England (formally the Health Protection Agency) and was developed in collaboration with teachers and stakeholders from 18 partner countries across Europe [11,12]. The European Directorate General funded its development for Health and Consumer Protection. Since the official launch in 2009, e-Bug has expanded across the world and, at present, has partners in 21 countries, including countries outside the European Union such as Turkey and Saudi Arabia. The resources are currently available in 22 different languages.

The e-Bug resources are hosted on the e-Bug website [13] and are all freely available for educators and students to download. The website is divided into two main sections: an area for teachers, and a separate area for students. Both areas are subdivided into junior and senior sections, for children ages 7-11 and 12-15 years, respectively. A third “Science Show” section hosts resources for children ages 5-7 years. The teacher area hosts lesson plans, activities, and worksheets covering 10 different topics, including microbes, spread of infections, antibiotics, and vaccines. The student area has Internet games and interactive activities for students to carry on learning at home.

Google Analytics has been used since 2010 to monitor Web traffic to the e-Bug website. Google Analytics has been used successfully to analyze Internet-delivered health interventions [14], and several publications have described the advantages and disadvantages to using this free service [15,16]. In addition to monitoring general website usage, changes in Web traffic can also be used to gauge the effect and success of public health awareness campaigns [17].

The aim of this report is to analyze the usage of the e-Bug website in order to understand how users access the website, where and when they access the site, and to review variation in use across the different areas of the site. This data will provide valuable information and can inform which pages should be improved and which could be promoted.

## Methods

Website statistics for the e-Bug website are collected, stored, and maintained by Google Analytics.

The code for collating weblogs by Google Analytics was added into the e-Bug website from March 2010. The code allows information to be collected by the Google software, which is then filtered out by country and stored under specific country profiles created in the Google Analytics software.

For the purpose of this report, we will present results from three school academic years between September 2010 and August 2013, with each year running from September 1 to August 31. Throughout the time period covered in this report, new language websites were added to e-Bug. Weblog data for these websites were collected from the first day the pages were created.

Google Analytics was used to gather data on the number of users, number of visits, location of visitors, visit duration, bounce rate, pages visited, source of access, and downloaded files. Where number of visitors is described in this report, the figure stated is the number of unique visitors to the website and does not include returning visitors. Google Analytics defines bounce rate as the percentage of single-page sessions.

## Results

### General Use of the Website

Between September 1, 2010 and August 31, 2013, the e-Bug website had 249,749 users and 324,601 visits from 211 different countries across the world. The number of users has increased in each academic year (September-August) (Table 1), with a 9.64% (6866/71,237) increase in users between 2010/2011 and 2011/2012 and a 28.56% (22,308/78,103) increase in users between 2011/2012 and 2012/2013. From the total number of users across the three academic years, 76.94% (249,749/324,601) of those are new users and 23.06% (74,852/324,601) are returning users.

The bounce rate and visit duration has also improved each year since September 2010. As shown in Table 1, the bounce rate decreased every year and the visit duration for each user increased.

**Table 1:** The number, duration, and bounce rate of visitors to the e-Bug website over the last three academic years.

Year	Number of visitors	Increase in visitors from previous year, n (%)	Visit duration (minutes)	Bounce rate (%)	*P* value (increase from previous year)
2010/2011	71,237	-	4.28	36	-
2011/2012	78,103	6866/71,237(9.63)	4.51	33	.74
2012/2013	100,410	22,308/78,103 (28.56)	5.22	31	.69

### General Use of the Website: Country Specific

**Figure 1:**
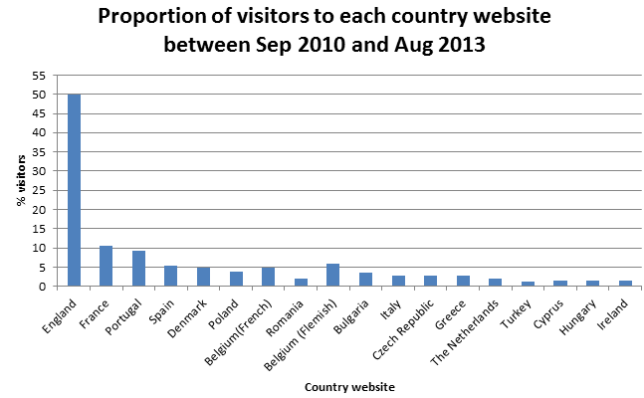
The proportion of visitors to each country's e-Bug website between September 2010 and August 2013.

The e-Bug website is currently available in 22 different languages, each having its own Web page which can be accessed from a menu on the home page. The English website is the most visited, with 50.45% (125,990/249,749) of all users over the three academic years, from 201 different countries, using the English site. The French and Portuguese websites are also visited frequently, with 10.53% (26,288/249,749) and 8.81% (22,013/249,749) of the users, respectively. Figure 1 shows the proportion of visitors to each country’s website over the three academic years.

### General Use of the Website: Seasonal Specific

**Figure 2:**
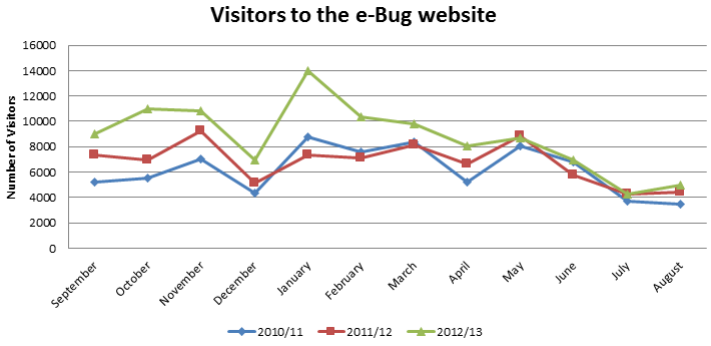
The number of visitors to the e-Bug website over each academic year.

The website is accessed most during the term times of the academic years. There is always a decrease in the number of users during the Christmas (December), Easter (April), and summer holidays (July-September), and Figure 2 shows this trend clearly. In each academic year, the website gets the most visits after Christmas during January and February.

e-Bug partners across Europe often run campaigns to promote and highlight the e-Bug resources. These campaigns are particularly effective when run in line with international health campaigns, such as EAAD or Global Hand Washing Day (GHD). For example, the EAAD website hosted links to the e-Bug website during the campaign in November 2012 [18]. The e-Bug website was also hosted on the GHD website during the campaign in October 2012 [19].

For GHD in October 2012, e-Bug worked in partnership with Schools Council UK to break a Guinness World Record for the largest simultaneous hand hygiene lesson at multiple venues. The event took place across the United Kingdom, with 22 schools and 2617 participants involved. The achievement of the Guinness World Record was announced on the e-Bug website in January 2013. Between October 1, 2012 and January 31, 2013, the period during which the hand washing campaign ran, the e-Bug website had 41,643 users, which was an increase of 48.90% (13,676/27,967) from the corresponding time period the year before (27,967 users). Despite the average annual increase in users each year, the additional increase over this time period is likely due to the hand washing campaign.

The majority of visitors to the e-Bug website, 58.43% (189,653/324,601) of all visits over the three academic years, access the site through organic search engines, with Google being the most popular among users. The majority of search terms are a variation on the word “e-Bug”, such as “ebug” or “e bug”, suggesting those users are looking directly for the e-Bug website and should therefore be classed as direct visitors. Other popular search terms which bring visitors to the e-Bug website include “useful microbes” and “harmful microbes”. After organic search engines, 25.58% (83,048/324,601) of visits access the site directly through the URL, with the remaining using referral websites such as the Health Protection Agency website or the Times Educational Supplement teacher resource website.

### Visits From Around the World

The majority of e-Bug users are based in the United Kingdom. Over the three academic years since September 2010, visits from the United Kingdom have totaled 36.17% (117,409/324,601) of all visits, with visits from France making up 10.94% (35,502/324,601) of the total. A list of the top 10 countries visiting e-Bug can be seen in Table 2. e-Bug has partners in 24 countries who promote e-Bug to schools, however, Table 2 shows that even without an active partner, e-Bug is still used in many countries across the world. From the top 10 countries visiting e-Bug across the three academic years, there are no active partners in Brazil, United States, or India.

In total, 211 different countries accessed the e-Bug website over the last three academic years. The majority of these visits (77.52%, 251,630/324,601) came from within Europe, as shown in Table 3. After Europe, e-Bug is then used mostly in the Americas (10.58%, 34,334/324,601), followed by Asia (6.30%, 20,448/324,601), and then approximately equally in Africa and Oceania (2.40%, 7778/324,601, and 2.12%, 6886/324,601, respectively).

Analysis of the Web statistics data shows that visitors from Brazil commonly use the Portuguese e-Bug website. In the last academic year (2012/2013), there were more visits to the Portuguese website from Brazil than there were from Portugal, 3751 visits from Brazil compared with 2589 from Portugal, although this may be accounted for by the larger population in Brazil. The United States and India both access the English e-Bug website.

**Table 2:** Top 10 countries visiting the e-Bug website between September 2010 and August 2013.

Country	Number of visits	% of total visits, n (%)
United Kingdom	117,409	117,409/324,601 (36.17)
France	35,502	35,502/324,601 (10.94)
Portugal	15,096	15,096/324,601 (4.65)
Denmark	13,659	13,659/324,601 (4.21)
Brazil	12,303	12,303/324,601 (3.79)
Belgium	12,209	12,209/324,601 (3.76)
United States	9016	9061/324,601 (2.79)
Germany	8919	8919/324,601 (2.75)
India	8778	8778/324,601 (2.70)
Greece	8517	8517/324,601 (2.62)

**Table 3:** Visits to the e-Bug website from continents across the globe between September 2010 and August 2013, and split by academic year.

Continent	Number of visits (2010-2013)	% of total visits, n (%)	% of total visits split by academic year, n (%)		
			2010/2011	2011/2012	2012/2013
Europe	251,630	251,630/324,601 (77.52)	76,155/92,893 (81.98)	75,924/100,058 (75.88)	99,551/131,650 (75.62)
Americas	34,334	34,334/324,601 (10.58)	8244/92,893 (8.87)	12,357/100,058 (12.35)	13,733/131,650 (10.43)
Asia	20,448	20,448/324,601 (6.30)	4819/92,893 (5.19)	5857/100,058 (5.85)	9772/131,650 (7.42)
Africa	7778	7778/324,601 (2.40)	1925/92,893 (2.07)	2783/100,058 (2.78)	3070/131,650 (2.33)
Oceania	6886	6886/324,601 (2.12)	1443/92,893 (1.55)	2378/100,058 (2.38)	3065/131,650 (2.33)

### The Most Commonly Used Resources

The e-Bug website is split into a teacher and student section, which are both further subdivided into areas for junior and senior students. Our analysis has identified that the teacher website has more users than the student website. In the 2012/2013 academic year, the most visited page in the teacher section was the “junior-e-Bug lesson pack” page, which contains the complete junior e-Bug resource pack for download, either in Word or PDF format, as well as links to each individual topic covered in the resource. This page had 19,055 visits. The most visited page in the student section was the “junior-Doctor Doctor” game with 10,820 visits.

For the English e-Bug Web pages, the junior sections in both the teacher and student areas are used more than the senior sections, with almost 4 times the number of visits to the most visited teacher pages for each section in the last year (19,055 visits compared with 5847 for senior), and 5 times more visits for the student section (10,820 visits compared with 2122 for senior). Interestingly, this trend in visits is not replicated for the French website, as here the senior website has slightly more visits than the junior website for both the teachers section and the student section. In the last year, the most visited French pages had 3259 visits for junior teacher and 4039 for senior teacher, with 566 for junior student and 633 for senior student.

The most popular topics used by teachers in all countries are “Introduction to Microbes”, “Useful Microbes”, “Harmful Microbes”, “Food Hygiene” (for junior students only), and “Hand Hygiene”. The “Farm Hygiene” resource (junior only) is used the least, most likely due to farm visits occurring mainly in the summer months, so overall usage is lower than other resources. Furthermore, not all schools visit farms and therefore fewer schools are likely to visit this page. The “Antibiotics” resource, for both junior and senior students, also has a low number of visitors.

For students, the most popular resources are the games, with “Doctor Doctor”, “Chicken Surprise”, and “Super Sneezes” being used the most. The “Disease Fact Files” and “Quiz” are also popular, although the “Revision Guides” and “Hall of Fame” rarely appear in the top 25 visited sections for both the junior and senior student website.

From the e-Bug Web pages, teachers can download all the resources to use within the classroom. The resources can either be downloaded as a complete pack, which contains all topics, or the individual lesson plans can be selected. Since September 2010, over 8000 complete resource packs have been downloaded from the e-Bug website, with almost 6000 of these being the junior pack. In total, there have been 267,910 downloads from the e-Bug website in the last 3 academic years. Table 4 shows these figures and identifies that almost half these downloads occurred in the 2012/2013 academic year.

**Table 4:** Total number of downloaded documents from the e-Bug website for each academic year.

Year	Number of downloads
2010/2011	52,673
2011/2012	73,473
2012/2013	141,774

## Discussion

### General Use of the Website

There has been a significant increase in the number of visitors to the e-Bug website over the last three academics years, demonstrating the need for educational resources on hygiene and infection. Between September 2010 and August 2013, the number of visitors increased by 40.95% (29,173/71,237). This increase is likely due to new international partner countries promoting e-Bug across the world, and to continued dissemination and promotion in the United Kingdom.

de Quincey et al [20] evaluated the usage of the e-Bug website between January 2008 and November 2009 using an application named Sawmill. During this time period, the website had >88,000 visitors, >169,000 downloaded documents, and an average of 3844 visitors per month. Although this raw data were cleaned before analysis with Sawmill, unlike the Google Analytics data presented here, we can still see that compared to the last year of data collection, between September 2012 and August 2013, the rate has more than doubled to an average of 8367 visitors per month.

The website statistics show that the e-Bug website is used throughout the academic year, with a decrease in the number of visitors during Christmas, Easter, and summer. This is expected as schools in Europe are closed and educators are on vacation. We can assume that users visiting the website during these holiday periods are mainly educators planning lessons for the next academic term, as the top five visited pages during these time periods are always on the teacher website. It was expected that visits to the student website would have increased during vacations, with students having more free time, however, our analysis has demonstrated that student visits also decrease during this time. It is therefore possible that student visits during term time may occur as homework related activities or as a result of being shown the website in schools.

The junior sections of the English website receive more visits than the senior sections, perhaps reflecting the number of junior versus senior schools in the United Kingdom, as of January 2014, according to the national statistics, there are five times more junior schools than senior schools [21]. This trend in visits is not seen in the French e-Bug website data, however, with the senior pages receiving slightly more visitors than the junior pages, despite there also being five times more junior schools than senior schools in France. The same pages and resources are visited most in both countries. This is likely to be due to the differences in the promotion and dissemination of e-Bug in France. The French Ministry of Education implements and promotes e-Bug in senior schools through a central mailing list; whereas junior schools are funded by their specific municipality, so they are more difficult to disseminate information to. More research is required to understand this difference in website usage.

January and February sees the most visitors to the website, which may be due to the cold and flu season, with schools wanting to highlight good hygiene practices to their students. This visit pattern is specific to England and France and is likely due to increased health promotion during the flu season and infections over the winter. The majority of overall visitors to the e-Bug website are from England and France (61.55%, 153,716/249,749), so these national campaigns will influence the overall visitors to the e-Bug website during January and February.

The Guinness World Record attempt may have contributed to the high number of visitors during January and February 2013. Visits during this time period were to all e-Bug resources, not just the hand hygiene pages, suggesting that campaigns like this encourage visits to other areas of the website. It is necessary to explore further whether e-Bug topics are generally taught in the winter term at school, in line with the National Curriculum, as this could affect the time of year that the website is visited and resources are downloaded.

Around 84.01% (272,701/324,601) of visitors to the e-Bug website access the site directly from the URL or by using search engines such as Google. The remaining 15.64% (50,756/324,601) visit the site from referral websites that link to e-Bug, suggesting that cross referencing or cross promotion of websites through similar sites is an important promotional tool.

### Visits From Around the World

The e-Bug resources are used mainly in the United Kingdom, but also across Europe and the rest of world. While the number of visits from Europe has been increasing, we also see more new visitors to e-Bug from outside Europe, in particular the Americas and Asia. This confirms that e-Bug’s reach is increasing across the globe. Even in countries where there has been no active dissemination of e-Bug via a partner, the website is still receiving visits. For example, the United States and India are frequently among the top 10 countries accessing the website. Having the website in languages used in more than one country has also helped increased global reach, for example, the high number of visitors in Brazil accessing the Portuguese website.

Monitoring the website statistics can give valuable information on how the website is used and the results can help inform future development and dissemination plans. The large number of visitors from Brazil suggests that there is a want by the users in that country for a health education resource of this type. Finding a partner to promote e-Bug in Brazil would ensure the resources are disseminated to as wide an audience as possible.

### The Most Commonly Used Resources

It is clear from our analysis that the student website is poorly visited, with considerably fewer visits than the teacher website. To increase use, future e-Bug development may focus around new resources and tools for this group, such as games and videos. The current student resources should be further evaluated to understand what changes are necessary in order to increase use, particularly the “Revision Guides” and “Hall of Fame” resources which are used the least, only appearing once in the top 25 visited junior student pages. This lack of visitors to the “Revision Guides” is likely to be a result of the already hugely popular revision resources available to students and teachers that are linked more closely to the National Curriculum. Examples include “BBC Bitesize” and “s-cool”. Promotion and dissemination of these less visited student resources should also be increased.

The most visited sections of the website are the teaching resource pages on “Introduction to Microbes”, “Useful Microbes”, “Harmful Microbes”, “Food Hygiene”, and “Hand Hygiene”. These should continue to be promoted to both junior and senior schools. Poorly visited resources, such as the “Farm Hygiene” and “Antibiotics” topics, should be more widely promoted. Previous research has shown that antibiotics are not widely included in the National Curriculum of many countries, unlike other e-Bug topics, which may explain their lower use [22]. This also suggests that the curriculum has a strong influence on how the e-Bug website is used.

e-Bug is mostly used in the United Kingdom and English-speaking countries, with 36.17% (117,409/324,601) of visitors based in the United Kingdom and over 50.45% (125,990/249,749) of visitors using the English website. Dissemination of the resources across the globe should increase in correlation with the increase in active partners and the increase in languages that e-Bug resources are available in. With 22 different language websites currently live, and more under translation, it is likely that more countries will be able to use and disseminate the resources to a wider audience.

### Limitations

Despite the usefulness of the Google Analytics data in tracking the use of e-Bug and visits to the website, there are also limitations. In order to fully interpret the results presented here, the data should be combined with qualitative data to understand users reasoning’s and behaviors. For example, the website statistics have shown that in the United Kingdom, the junior websites are visited more than the senior websites, and the teacher websites are visited more than the students websites. Qualitative data could provide an explanation as to this difference and help guide future development or adaptations to the resources.

In addition, it is hard to gain an accurate usage of the e-Bug resources from this data as the resources are downloadable, meaning that teachers can continue to use the resources without revisiting the website. The complete e-Bug teaching pack can also be downloaded, meaning each individual topic page does not need to be visited. In 2010, printed e-Bug teaching packs were also distributed to all schools in England, meaning that teachers had no need to visit the website. It is likely that usage of e-Bug is much higher than that which is predicted from the Google Analytics data. It would be useful to explore the usage of the printed e-Bug resources distributed to all schools in England, as this may have influenced the number of visitors to the e-Bug site in that same year. It would be recommended to use qualitative research to gather this information to determine whether receiving the e-Bug teaching packs prevented teachers from visiting the website.

While the number of visits to the website is an important measurement tool of the growth of e-Bug, it is also necessary to explore the outcome of the visit to the website. Qualitative research should be conducted to determine whether the visit, and subsequent teaching, resulted in outcomes such as improved knowledge, improved enthusiasm for the subject, change in student health behaviors, for example, hand washing techniques and change in teaching methods including health topic delivery.

### Implications

Results from the weblog analysis have found that qualitative work is needed to explore reasons for the poor use of the teacher “Farm Hygiene” and “Antibiotic” resources, and the student “Hall of Fame” resource and “Revision Guides”. Further promotion of these resources is needed. In promotional materials to schools and educators, e-Bug should continue to highlight the most popular resources such as the “Introduction to Microbes”, “Useful Microbes”, and “Harmful Microbes” topics. In addition, e-Bug needs to extend partnerships to other countries speaking the languages that are available. Finally, e-Bug should be promoted via links from other health and educational websites and other social media websites used by teachers and children.

## Abbreviations

EAAD: European Antibiotic Awareness Day
GHD: Global Hand Washing Day
NHS: National Health Service
WHO: World Health Organization
